# Biochemical characterization of ferric uptake regulator (Fur) from *Aliivibrio salmonicida*. Mapping the DNA sequence specificity through binding studies and structural modelling

**DOI:** 10.1007/s10534-020-00240-6

**Published:** 2020-07-09

**Authors:** Kristel Berg, Hege Lynum Pedersen, Ingar Leiros

**Affiliations:** Department of Chemistry, Faculty of Science and Technology, The Norwegian Structural Biology Centre (NorStruct), UiT the Arctic University of Norway, 9037 Tromsø, Norway

**Keywords:** Ferric uptake regulator, Metal binding, DNA interactions, Aliivibrio salmonicida, Electrophoretic mobility shift assay

## Abstract

Iron is an essential nutrient for bacteria, however its propensity to form toxic hydroxyl radicals at high intracellular concentrations, requires its acquisition to be tightly regulated. Ferric uptake regulator (Fur) is a metal-dependent DNA-binding protein that acts as a transcriptional regulator in maintaining iron metabolism in bacteria and is a highly interesting target in the design of new antibacterial drugs. Fur mutants have been shown to exhibit decreased virulence in infection models. The protein interacts specifically with DNA at binding sites designated as ‘Fur boxes’. In the present study, we have investigated the interaction between Fur from the fish pathogen *Aliivibrio salmonicida* (AsFur) and its target DNA using a combination of biochemical and in silico methods. A series of target DNA oligomers were designed based on analyses of Fur boxes from other species, and affinities assessed using electrophoretic mobility shift assay. Binding strengths were interpreted in the context of homology models of AsFur to gain molecular-level insight into binding specificity.

**Electronic supplementary material:**

The online version of this article (10.1007/s10534-020-00240-6) contains supplementary material, which is available to authorized users.

## Introduction

Iron is an essential nutrient for all living organisms and many key biological processes are dependent on its abundance. For bacteria, iron is crucial for growth and host colonization. Iron mostly exists in the insoluble Fe^3+^ form under aerobic conditions at physiological pH and availability of the soluble reduced form, Fe^2+^, is restricted. Due to the ability of free iron to form toxic hydroxyl radicals through the Fenton reaction (Guerinot 1994), the essential high-affinity uptake systems of iron and iron homeostasis in bacteria must be tightly regulated, and in most bacteria, these processes are under control of the global metalloregulator Ferric uptake regulator (Fur) (Hantke 2001). Fur was first described in *Escherichia coli* (Hantke 1981), where it controls the expression of more than 90 genes, and its chemical properties and role in homeostasis has since been studied in homologs from multiple bacteria, including *Mycobacterium smegmatis* (Gao et al. 2019), *Acidovorax citrulli* (Liu et al. 2019), *Campylobacter jejuni* (Sarvan et al. 2019), *Porphyromonas gingivalis* (Smiga et al. 2019) and *Salmonella enterica* serovar Typhimurium (Wang et al. 2019). Although Fur was originally described as a repressor of genes coding for components of the ferric uptake systems found in the cell membrane, it is now understood to control the expression of toxins such as hemolysin and exotoxins, as well as proteins involved in iron-scavenging and uptake systems (Prince et al. 1991; Vasil and Ochsner 1999).

The typical model of action states that when intracellular levels of iron are high, dimeric Fur will act as a repressor by complexing Fe^2+^, binding specific Fur recognition sites in the promoter region and preventing transcription of associated genes involved in iron uptake, storage and metabolism. Similarly, when iron is limiting, the Fe-Fur complex dissociates from the promoter and allows gene expression. Recent studies however, have broadened our understanding of Fur-mediated regulation, indicating that Fur also may function as an activator and act in an iron-independent manner (Butcher et al. 2012; Miles et al. 2010), for example in *Helicobacter pylori*, all four combinations of Fur regulation have been characterized: repression and activation, with or without cofactor (Carpenter et al. 2009; Danielli and Scarlato 2010). Further, apo-Fur repression has been described in *Staphylococcus aureus* (Deng et al. 2012) and *Campylobacter jejuni* (Grabowska et al. 2011).

In addition to iron, which is the primary functional metal bound in vivo, DNA-binding by Fur can be activated by other divalent metals in vitro; Mn^2+^, Cu^2+^, Cd^2+^, Ni^2+^, Co^2+^ or Zn^2+^ (Bagg and Neilands 1987; de Lorenzo et al. 1987; Gao et al. 2008; Mills and Marletta 2005; Ochsner et al. 1995). Mn^2+^ is considered a suitable physiological mimic of Fe^2+^ for in vitro studies as it is bound with a similar affinity to Fe^2+^ by *E. coli* Fur and adopts the same hexacoordinated octahedral geometry using conserved residues, as seen in recent metal-bound crystal structures of Fur from *Francisella tularensis* (Deng et al. 2015; Mills and Marletta 2005; Perard et al. 2018)*.* In contrast Zn^2+^ is bound with lower affinity and in a tetrahedral geometry (Deng et al. 2015; Perard et al. 2018)*.*

Fur enacts its biological DNA-binding function as a dimer (Michaud-Soret et al. 1997), but may exist in several oligomeric states in solution, depending on protein concentration, salt concentration and pH (D'Autreaux et al. 2007). Each Fur monomer consists of two domains; an N-terminal winged helix-shaped domain involved in DNA binding (DNA binding domain; DBD) and a C-terminal α/β domain involved in protein dimerization (Dimerization domain; DD) (Supplementary Figure S1) (Hernandez et al. 2005; Pohl et al. 2003; Stojiljkovic and Hantke 1995). Crystal structures of apo- and holo-Fur have been available for some time from several bacterial species including *Pseudomonas aeruginosa* Fur (Pohl et al. 2003)*, F. tularensis* Fur (Perard et al. 2018), *Vibrio cholerae *Fur (Sheikh and Taylor 2009), *H. pylori* Fur (Dian et al. 2011), *C. jejuni* Fur (Butcher et al. 2012), as well as a crystal structure of the DBD of *E. coli* Fur (Pecqueur et al. 2006); however, only with the recent structures of *Magnetospirillum gryphiswaldense* MSR-1 Fur (MgFur) in complex with DNA have structural details of Fur-DNA interactions become clear (Deng et al. 2015). A series of crystal structures, which include apo-Fur, holo-Fur and two different Fur-DNA complexes, gave a better understanding of issues regarding metal-binding, molecular mechanisms and structural basis of Fur-DNA interaction, at least for that organism.

The Fur-DNA interaction site, generally referred to as the “Fur box”, is a conserved sequence motif represented by a 19 base pair (bp) palindrome, located between the -35 and -10 sites at the promoters of Fur-regulated genes. The classical Fur box (Supplementary Figure S2a) originates from DNase I protection- and footprinting-experiments on *E. coli* Fur, where a Fur dimer recognizes a 19 bp inverted repeat sequence: 5′-GATAATGATAATCATTATC-3′ (Escolar et al. 1999), although this exact sequence is not found in the *E. coli* genome. This inverted repeat operator site was confirmed by binding of Fur to oligonucleotides inserted into a plasmid (Calderwood and Mekalanos 1988). In addition, Fur boxes from other genera have also been characterized and described (Baichoo and Helmann 2002; Escolar et al. 1998, 1999, 2000; Pedersen et al. 2010; Pich et al. 2012). While a recapture of the alternative arrangements of the Fur box and its interaction with Fur can be found in Supplementary text and Supplementary Figure S2, the binding modes observed from crystal structures of MgFur in complex with Fur box mimics are consistent with: a 9-1-9 inverted repeat model (Supplementary Figure S2a), where one MgFur dimer interacts; and a 7-1-7 heptamer inverted repeat model (Supplementary Figure S2e), where two MgFur dimers interact with DNA. The latter is consistent with a slightly extended Fur consensus sequence of 21 bp (Supplementary Figure S2e). Crystal structures determined for complexes of DtxR bound to its operator site show a similar binding model (Pohl et al. 1999; White et al. 1998).

In the search for the shortest recognition unit by Fur, the 7-1-7 inverted repeat was found to be the minimum in *B. subtilis* Fur (Baichoo and Helmann 2002). Single 6-mer or 7-mer oligonucleotides showed no affinity to Fur and Fur boxes with two 6-mers bound weakly. Similar results were obtained for *E. coli* Fur, where a minimum of three repeats of the hexameric motif GATAAT was required for Fur binding (Escolar et al. 2000). Thus, in the search for a Fur box consensus, the focus is shifting towards the functional pattern within the sequence, rather than the specific sequence or length. The consensus hexamer NATA/TAT appears to be the main unit of interaction with Fur, regardless of orientation and number. In addition, Fur boxes typically have a high content of A/T bases. Experimentally and computationally determined Fur boxes in various bacteria showed consensus sequence identity ranging from 50 to 80%. (Ahmad et al. 2009a; Baichoo et al. 2002; Pedersen et al. 2010; Sebastian et al. 2002; Thompson et al. 2002), and Fur appears to have a rather broad substrate-binding ability.

The published crystal structures of MgFur in complex with two different DNA targets demonstrate the lack of a well-defined sequence specificity, and a high degree of degeneration in the Fur box (Deng et al. 2015). DNase I footprinting with the *feoAB1* operator showed a protected region without the typical arrays of GATAAT hexamers. However, for successful co-crystallization, the *feoAB1* operator was mutated to a near-perfect inverted repeat, which bound one dimer of MgFur with similar binding affinities to the original *feoAB1* operator. Gel shift-based assays showed that MgFur also specifically binds the *P. aeruginosa* Fur box, and furthermore, that two dimers of MgFur co-crystallized with the *P. aeruginosa* Fur box sequence (identical in sequence to the *E. coli* Fur box, which we will use throughout) (Deng et al. 2015). These MgFur-DNA complex structures are the first to demonstrate the ability to bind DNA at different ratios.

Common for these two rather different DNA targets is the way each Fur monomer formed contacts with both DNA strands using its DNA-binding domain (DBD), which interacted with a 10–11 bp sequence containing an important G base, conserved T base and an AT-rich region characterised by a narrow minor groove. In vivo experiments indicated that specific Fur-DNA contacts may be directly connected to DNA shape instead of being base specific. The positively charged Lys15 in MgFur bound this narrow minor groove with enhanced negative electrostatic potential. The narrow minor groove of AT-rich sequences is highlighted as an essential feature for Fur interaction (Deng et al. 2015).

The Gram-negative Vibrionaceae family of gamma-proteobacteria include many mammalian pathogens, and the role of Fur and iron homeostasis in infection has received much attention due to its potential as a drug target (Jones and Oliver 2009; León-Sicairos et al. 2015; Mey et al. 2005; Wright et al. 1981). Amino acid alignments and phylogenetic analysis shows that the Fur protein is highly conserved within the Vibrionaceae, and in the present study we have investigated Fur from the Vibrio fish pathogen *Aliivibrio salmonicida*, the causative agent of cold-water vibriosis in Atlantic salmon and cod (Egidius et al. 1986). Previously, Thode et al. demonstrated a key role of *A. salmonicida* Fur (hereafter AsFur) in iron homeostasis (Thode et al. 2017) where construction of a *fur* null mutant strain severely affected fitness and growth of the bacteria, caused oxidative stress and a general reduced ability to cope with low-iron conditions. Furthermore, evaluation of expression levels compared to the wild-type identified up-regulation of numerous genes encoding for iron uptake and storage and down-regulation of potential targets for RyhB and other sRNAs involved in iron homeostasis (Thode et al. 2017). AsFur and its DNA target (Fur box) have previously been studied in vitro and in silico, with emphasis on identification of residues of importance for protein-DNA interactions (Ahmad et al. 2009b; Pedersen et al. 2010). A 19 bp inverted repeat Vibrio Fur box consensus, 5′-AATGATAATAATTATCATT-3′, was identified by computational methods (Ahmad et al. 2009b) and later shown to be specifically recognized and bound by AsFur in vitro in EMSA experiments with strong affinity (Pedersen et al. 2010). Additionally, specific individual nucleotides and amino acid residues possibly interacting in the AsFur-Vibrio Fur box complex have been predicted, some species-specific for AsFur. In the Vibrio Fur box, A14, C16 and T13 were suggested to contribute directly to the AsFur-DNA complex, on one or both strands. By homology modelling, the C16 nucleotide was predicted to be in close proximity to the amino acids Tyr56, Arg57 and Arg70, identified by binding free energy calculations (Pedersen et al. 2010). However, the base-specificity of these interactions remained elusive.

While these previous studies have mainly focused on investigating the effect of amino acid substitutions on DNA interaction, the present study aimed to elucidate the effect of nucleotide substitutions in the target DNA in an attempt to establish the binding mode of AsFur on Fur box-DNA.

In this study, we have characterized AsFur with respect to activity, thermal stability and its binding capability on a range of oligonucleotides in order to investigate the importance of key nucleotides in AsFur-DNA interaction.

## Materials and methods

### Cloning, expression and purification of AsFur

AsFur was overexpressed and purified with some changes from the previously described protocol (Pedersen et al. 2010)*.* Following cloning of the *fur* gene from *A. salmonicida* into the pDEST14 Gateway expression vector (Invitrogen™, USA), AsFur was overexpressed at 20ºC overnight in *E. coli* BL21-CodonPlus® (DE3)-RIL competent cells, grown in LB broth with 100 µg/ml ampicillin and 34 µg/ml chloramphenicol. Harvested cells were resuspended in lysis buffer (Buffer A; 300 mM NaCl, 50 mM Tris–HCl, 5 mM β-mercaptoethanol, 10 mM Imidazole, pH 7.5). The histidine-rich AsFur was purified by Immobilized metal ion affinity chromatography (IMAC) on a 5 ml HisTrap HP column (GE Healthcare). Buffer A was used as the running buffer and Buffer B (300 mM NaCl, 50 mM Tris–HCl, 5 mM β-mercaptoethanol, 500 mM Imidazole, pH 7.5) as the elution buffer. The second purification step was performed using size-exclusion chromatography (SEC) on a Superdex 200 16/60 gel filtration column (GE Healthcare) equilibrated with Buffer A without Imidazole added. AsFur purity was verified by SDS-PAGE and protein concentration was determined by NanoDrop 2000c (Thermo Scientific) using the theoretical extinction coefficient.

### Thermofluor

In order to improve the stability of the purified AsFur, thermal stability in various buffer systems and salt concentrations was investigated by a Thermofluor assay (Ericsson et al. 2006). Protein unfolding and its melting temperature (T_m_) is monitored by using the fluoroprobe SYPRO Orange dye which emits fluorescence upon binding to exposed hydrophobic regions.

The buffer screen contained 24 buffers covering a pH range from 4.5 to 9.0. Briefly, 5 µl protein (2.5 mg/ml), 12.5 µl 2 × buffer solution (100 mM) and 7.5 µl 300 × SYPRO® Orange (Sigma Aldrich) were mixed and added to the wells of a 48-well PCR-plate (Bio-Rad). To assess the effect of various salts, 15 µl of protein (0.8 mg/ml) diluted in the appropriate buffer (Tris pH 7.5) were mixed with 7.5 µl of 300 × SYPRO® Orange (Sigma Aldrich) and 2.5 µl different salts in concentrations ranging from 0.1–2.0 M. The plates were sealed with Microseal® 'B' Adhesive Seals (Bio-Rad) and heated in a MiniOpticon Real-Time PCR System from 20 to 80 °C in increments of 1 °C per sec. Melting curves were monitored with a charge-coupled device (CCD) camera with wavelengths for excitation and emission at 490 and 575 nm, respectively. T_m_, corresponding to the midpoint of the transition curve, was determined using the supplied instrument software and monitoring the fluorescence of the HEX channel.

### DNA protection assay and the effect of metals on Fur binding

The capability of purified AsFur to bind DNA in the presence of various metals was investigated using a restriction site protection assay. The aerobactin plasmid pDT10, (kindly provided by Isabelle Michaud-Soret, Grenoble, France) carries four restriction enzyme sites, with the *E. coli* Fur box incorporated into one of the HinfI sites (D'Autreaux et al. 2002). Based on the method developed by Bagg and Neilands (Bagg and Neilands 1987), activated Fur binds the Fur box and thereby makes the restriction site unavailable for digestion by HinfI*.* Fur activity is confirmed by observing digestion patterns on gel electrophoresis. Fur is active if a 1781 bp band is observed, while two bands, respectively 1530 bp and 251 bp are observed if the protein is inactive. Supplementary Figure S3 (modified from (Cisse et al. 2014)) summarizes the principle behind the assay.

AsFur (20 µM) was incubated with two equivalents of a range of metals (40 µM) in binding buffer (100 mM BisTrisPropane pH 7.5, 100 mM KCl, 5 mM MgSO_4_) for 10 min at room temperature, followed by addition of pDT10 plasmid at 10 nM final concentration and 20 min additional incubation. Restriction enzyme digestion was carried out by adding 4 units per µl of HinfI to the mixture and incubating for 1 h at 37 °C before quenching with 0.5 mM of EDTA. The samples were run for 30 min at 100 V on 1% agarose gel in TAE and visualized under UV light.

### Design of synthetic Fur box-containing oligonucleotides

To investigate the effect of length and specific base-substitutions to the Vibrio and *E. coli* respective Fur boxes, a range of oligonucleotides of various lengths (15–24 nt) were designed. In short, single-stranded DNA (Sigma-Aldrich) were diluted in Buffer C (50 mM HEPES pH 8, 50 mM NaCl) to 1 mM and annealed to double-stranded DNA by boiling for 5 min and cooling slowly to room temperature. Annealed oligonucleotides were separated by anion-exchange liquid-chromatography column (Mono-Q), with Buffer C as running buffer and Buffer D (50 mM HEPES pH 8, 1 M NaCl) as elution buffer, followed by dialyses in Slide-A-Lyzer Dialysis Cassettes (3.5 k MWCO; Thermo Scientific) overnight back to Buffer C. DNA concentrations were measured by Nanodrop 2000c (Thermo Scientific).

### Electrophoretic gel mobility shift assay (EMSA)

Unlabelled Fur box-mimicking oligonucleotides were used as probes in EMSA assays, where DNA mobility is detected by the double stranded nucleic acid stain SybrGreen (Life Technologies), and slower mobility indicates that AsFur has complexed with the Fur box.

To complex AsFur with its DNA target, desired concentrations of purified AsFur were incubated with binding buffer (20 mM Tris acetate pH 8.0, 1 mM MgCl_2_, 50 mM KCl, 1 mM DTT and 100 µM MnCl_2_) at RT for 20 min. After addition of DNA (5 µM), the mixture was incubated for another 30 min before adding 10 × loading dye (30% glycerol in binding buffer). Samples were loaded on native 8% polyacrylamide/TB gels and electrophoresis was performed at 200 V for 2–2½ hours and at 6ºC with circulating 1 × TB buffer (89 mM Tris, 89 mM boric acid, pH 8.0). Finally, the gel was incubated with SybrGreen 1:10,000 in TB buffer for 20 min and band shifts were detected under UV light at ~ 254 nm. Binding strengths were examined and rated by visualization.

### Analysis of AsFur compared to functional and structural homologs

Homology models of AsFur were generated using as templates the crystal structures of MgFur in complex with the *E. coli* Fur box (PDB4rb1) and the *feoAB1* operator (PDB4rb3), respectively. The modelling tools of the Swiss-model repository were utilized in default mode to obtain the homology models. Both template structures contain Mn^2+^-ions in the regulatory S2 and structural S3 sites and were kept in the homology model. Although most likely present in AsFur, the structural S1 metal site generally coordinated by four Cysteine residues was not taken into account in the AsFur homology models as this site has a remote location to the DNA-interaction region. The two different interactions modes and stoichiometry of MgFur interacting with dsDNA were further analysed using WinCoot (Emsley et al. 2010) and visualized by PyMol (www.pymol.org). Conserved nucleotide base-protein interactions were highlighted from structure-based sequence alignments with homologous Fur crystal structures, rendered by ESPript 3.0 (Robert and Gouet 2014) and from the output from the NuProPlot server (Pradhan and Nam 2015).

## Results and discussion

### AsFur was purified to homogeneity through affinity- and size exclusion-chromatography

**Fig. 1:**
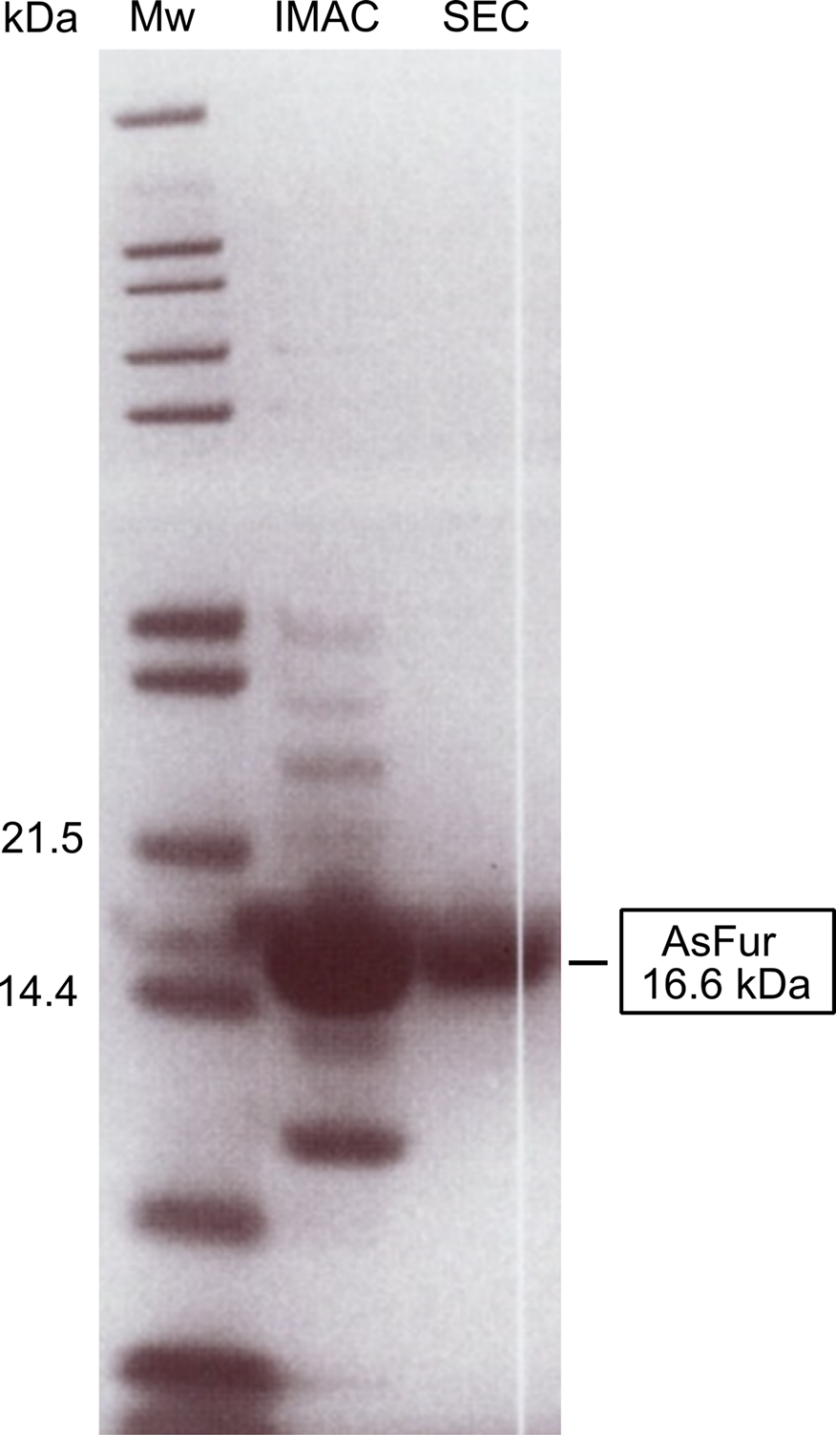
Coomassie Blue-stained SDS-PAGE showing molecular weight marker and the collected fractions from IMAC and SEC, respectively. The relevant molecular weights (Mw; kDa) are indicated

AsFur consists of 147 amino acid residues with theoretical pI and molecular weight of 5.75 and 16.6 kDa, respectively. A large-scale purification procedure of AsFur was established by Pedersen et al. (Pedersen et al. 2010). In brief, the *fur* gene from *A. salmonicida* was cloned, over-expressed in BL21-CodonPlus® (DE3)-RIL and purified to apparent homogeneity by two consecutive steps; IMAC affinity purification using HisTrap HP followed by SEC using HiLoad Superdex 200 pg. From SEC chromatography, AsFur fractions were detected at a volume corresponding to a homodimer, consistent with previous observations (Pedersen et al. 2010). The SDS-PAGE analysis of purified AsFur is shown in Fig. 1.

### Thermal denaturation screening on pH and salt showed a slight effect on the stabilization of Fur

**Fig. 2:**
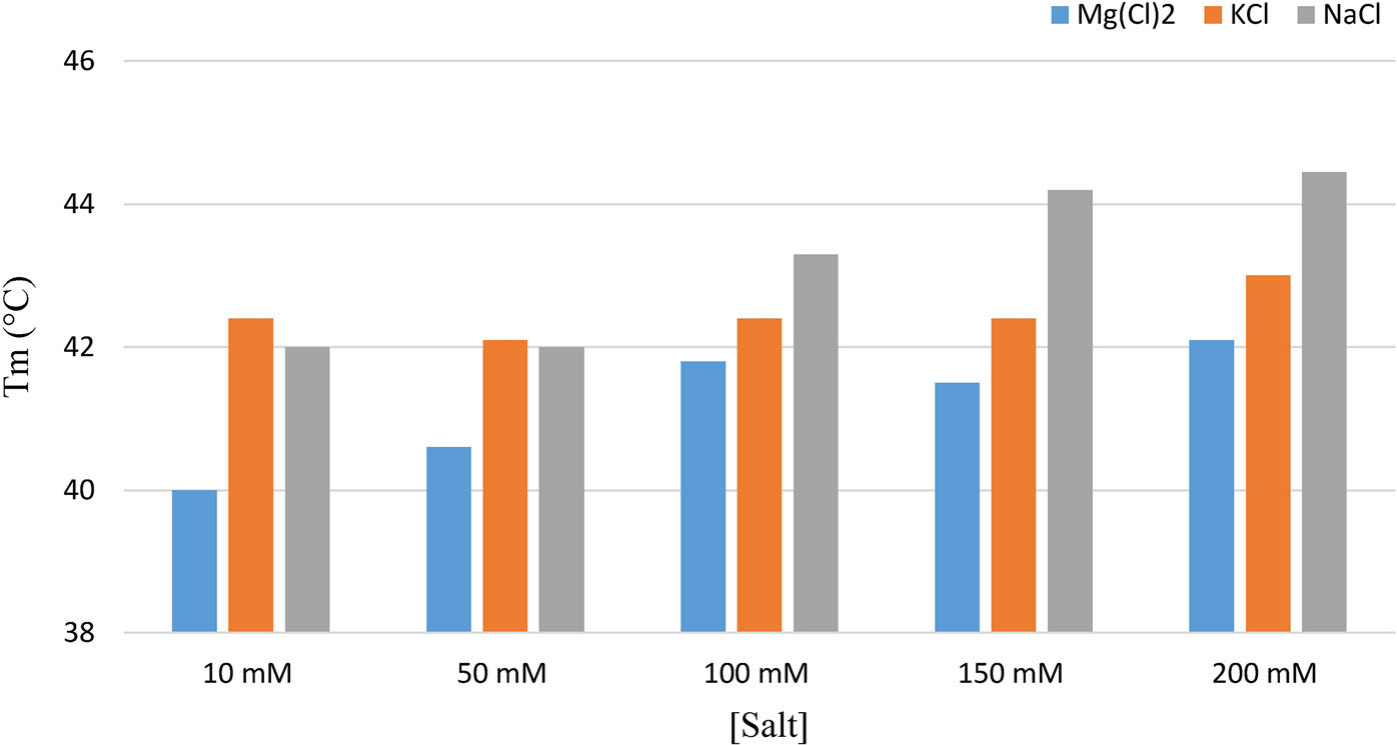
The effect of salt on thermostability. Standard buffer conditions were Tris–HCl, pH 7.5. (Color figure online)

Although AsFur was purified to homogeneity as seen in Fig. 1, the initial protein batches showed a tendency to aggregate, with complete loss of binding activity within a week at standard storage conditions. To avoid protein aggregation and increase stability, a thermal shift assay (Thermofluor) was implemented to identify better buffer conditions. Screening of a range of buffer compositions (and pH) by Thermofluor only showed negligible effects on AsFur stability, however, activity assays indicated that a minor change in pH from 8.0 to 7.5 in Tris-buffer reduced aggregation and increased the storage stability of AsFur at 4 °C. Furthermore, a slight increase in NaCl concentration up to 200 mM showed a positive effect on AsFur stability (Fig. 2), in comparison to MgCl_2_ and KCl where only minor improvements could be seen. Although the storage stability of AsFur was improved by the above-mentioned changes in pH and NaCl concentrations, batch variations were still a frequent problem in the following characterization.

### Presence of divalent metals alters DNA binding by AsFur

In the classical regulation pattern, iron is the primary functional metal that dimerizes and activates Fur in vivo*.* The ability of Fur to be effectively activated by a wide range of other divalent metal ions in vitro has prompted discussions about the true physiological metal responsible for Fur activation, although evaluation of Fur metal affinity by metal titration experiments suggests that only Fe^2+^ show sufficient affinity to activate Fur within relevant concentration ranges in vivo (Mills and Marletta 2005). However, elevated concentrations of other metals intracellularly could have implications for the normal iron regulation and the different metal bound Fur could potentially act on different DNA targets (Hantke 1987).

**Fig. 3:**
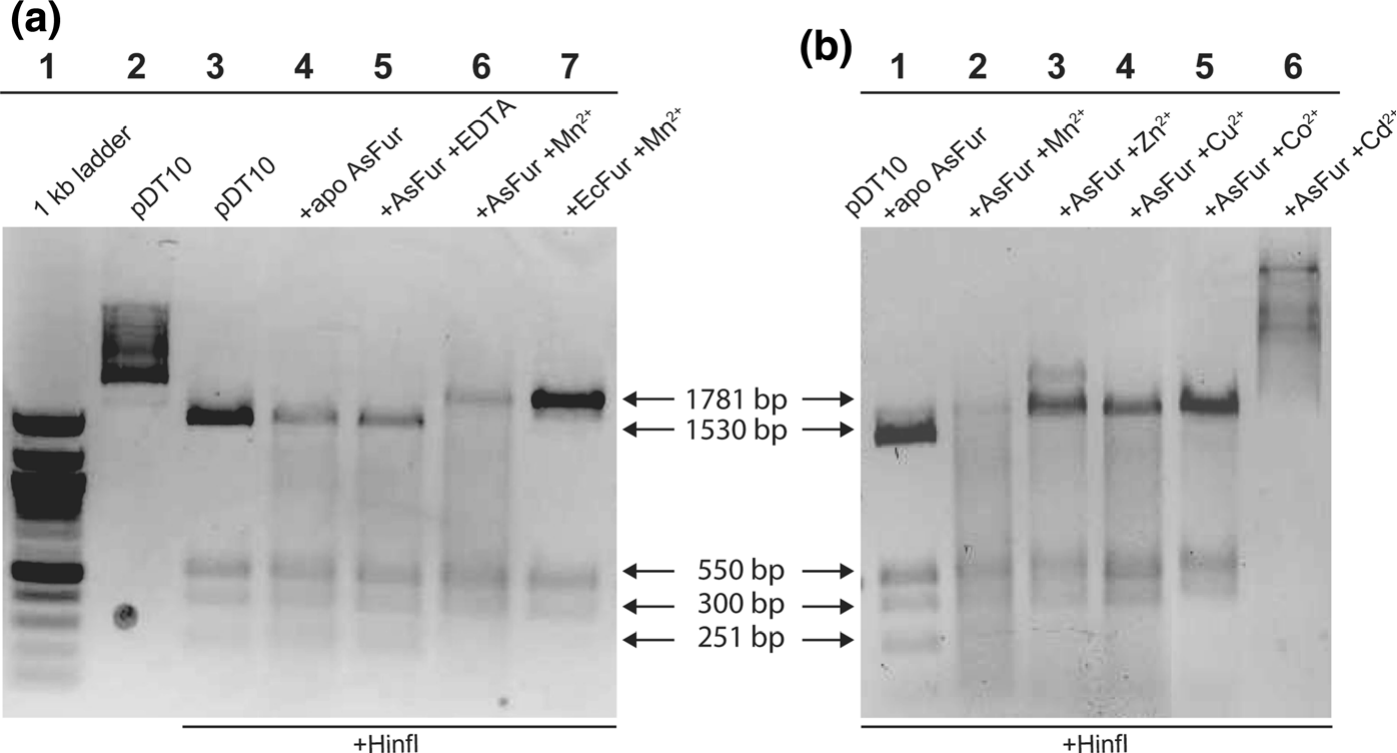
Plasmid protection assay verifying AsFur DNA binding in the presence of two equivalents of manganese (**a**) and identifying additional metals able to activate AsFur (**b**). The plasmid pDT10 was cleaved by HinfI in the absence or presence of active Fur and the digested migration pattern was analyzed by 1% gel electrophoresis. Active AsFur binds to the incorporated Fur box in the 1781 bp restriction fragment and protects it from being cleaved into 1530-bp and 251-bp fragments. **a** Lane 1: 1 kb ladder; Lane 2: plasmid pDT10; Lane 3: pDT10 + HinfI; Lane 4: pDT10 + apo AsFur + HinfI; Lane 5: pDT10 + AsFur + EDTA + HinfI; Lane 6: pDT10 + AsFur + Mn^2+^ + HinfI; Lane 7: pDT10 + EcFur + Mn^2+^ + HinfI. **b** Lane 1: plasmid pDT10 + apo AsFur + HinfI; Lane 2: pDT10 + As-Fur + Mn^2+^ + HinfI; Lane 3: pDT10 + AsFur + Zn^2+^ + HinfI; Lane 4: pDT10 + AsFur + Cu^2+^ + HinfI; Lane 5: pDT10 + AsFur + Co^2+^ + HinfI; Lane 6: pDT10 + AsFur + Cd^2+^ + HinfI

To measure the effect of a range of metals on AsFur-DNA binding, an in vitro assay utilising protection of a restriction site in the aerobactin promoter was used (D'Autreaux et al. 2002). Functional binding by Fur is envisaged by the absence of a fourth 251 bp band on the gel and an increase in size of the upper band to 1781 bp. Analysis showed that AsFur binds the aerobactin promoter in a metal-dependent fashion with Mn^2+^ present (Fig. 3a). Furthermore, the results in Fig. 3b show that AsFur also is able to bind the Fur box in presence of the divalent metal cations Mn^2+^, Zn^2+^, Cu^2+^ and Co^2+^. Fe^2+^ is considered the most physiologically-relevant metal ion for Fur activation, however it was omitted from this panel as its rapid oxidation precludes its use under standard assay conditions. Although plasmid protection appears weaker for Mn^2+^ compared to Zn^2+^, Cu^2+^ and in particular Co^2+^, Mn^2+^ was still the preferred choice for subsequent AsFur-DNA interaction studies, as Mn^2+^ and Fe^2+^ have been shown to have conserved metal coordination and similar chemical behavior in structural studies (Perard et al. 2018). The behaviour of AsFur in the presence of Cd^2+^ could not be interpreted, as the migration pattern resembles that of untreated plasmid, suggesting that HinfI is inhibited in the presence of Cd^2+^.

### Oligonucleotides of different lengths and sequences have an effect on the Fur-DNA binding

The 19 bp inverted repeat Vibrio consensus sequence, 5′-AATGATAATAATTATCATT-3′, as well as the *E. coli* Fur box, 5′-GATAATGATAATCATTATC-3′, formed the templates for EMSA assays of a range of oligonucleotides (see Table 1 for oligonucleotide composition). Single bases and/or arrays of bases were mutated to examine possible important binding sites or important hexamer arrangements. Binding strengths were examined and rated.

**Table 1:**
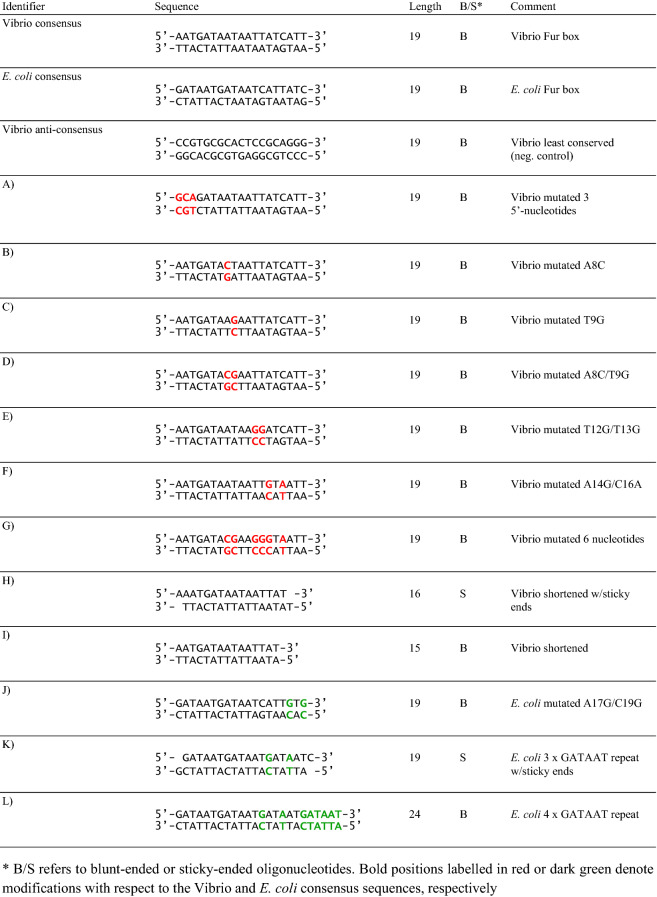
Oligonucleotides selected and tested for AsFur interaction by EMSA

**Fig. 4:**
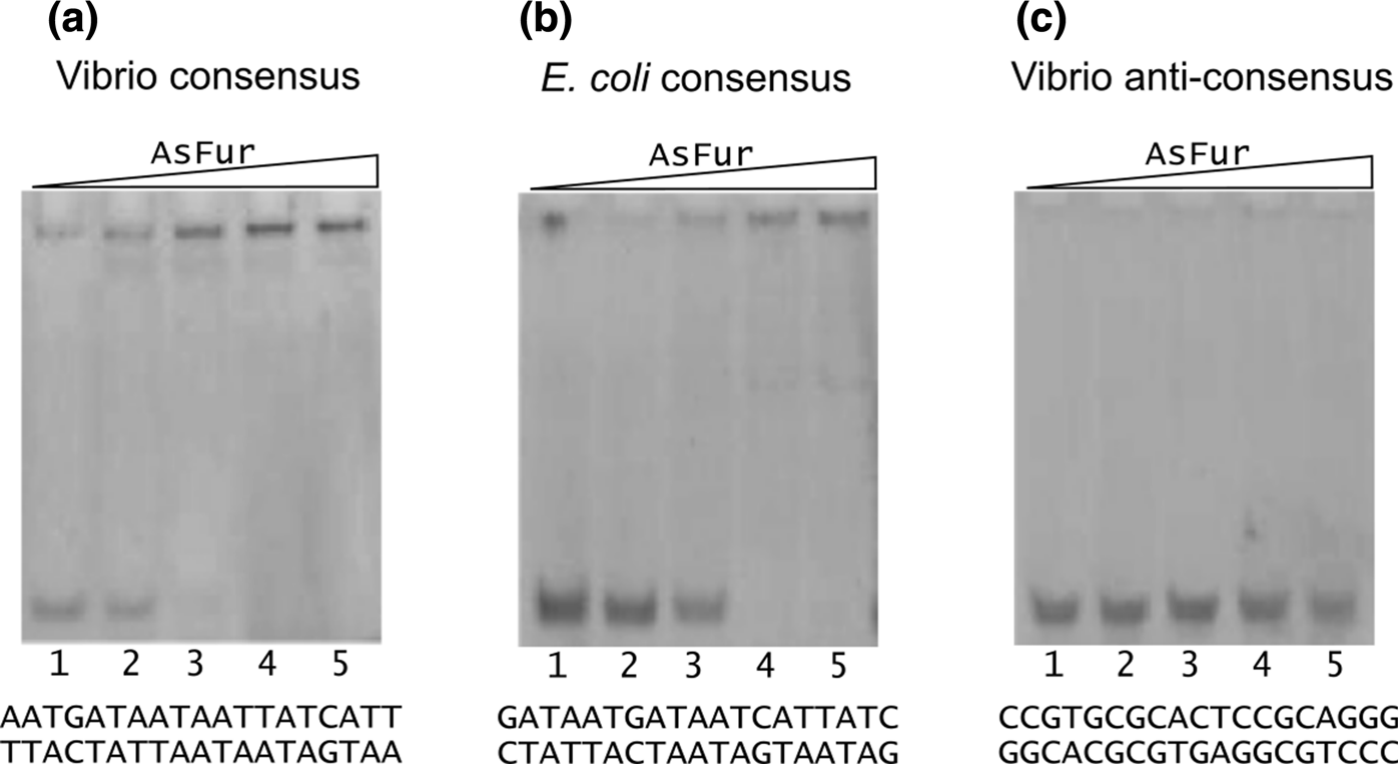
EMSA positive and negative controls. **a** Vibrio consensus sequence. **b** *E. coli* consensus sequence. **c** Vibrio anti-consensus sequence. 5 μM DNA was incubated with increasing concentrations of AsFur (0, 10, 20, 40 and 80 μM) for lanes 1–5, respectively. The experiments were performed in the presence of Mn^2+^

The oligonucleotides were loosely grouped based on conservation and length. First, EMSA experiments were performed in order to verify interaction of AsFur with the consensus sequences from both Vibrio species and *E. coli* using the least-conserved Vibrio sequence (Pedersen et al. 2010) as a negative control (Fig. 4). Strong interaction was observed with the Vibrio consensus sequence (Fig. 4a), and moderate binding was also seen with the *E. coli* consensus sequence (Fig. 4b), with essentially no interaction detected with the Vibrio least-conserved (anti-consensus) sequence even at the highest AsFur concentration (Fig. 4c).

**Fig. 5:**
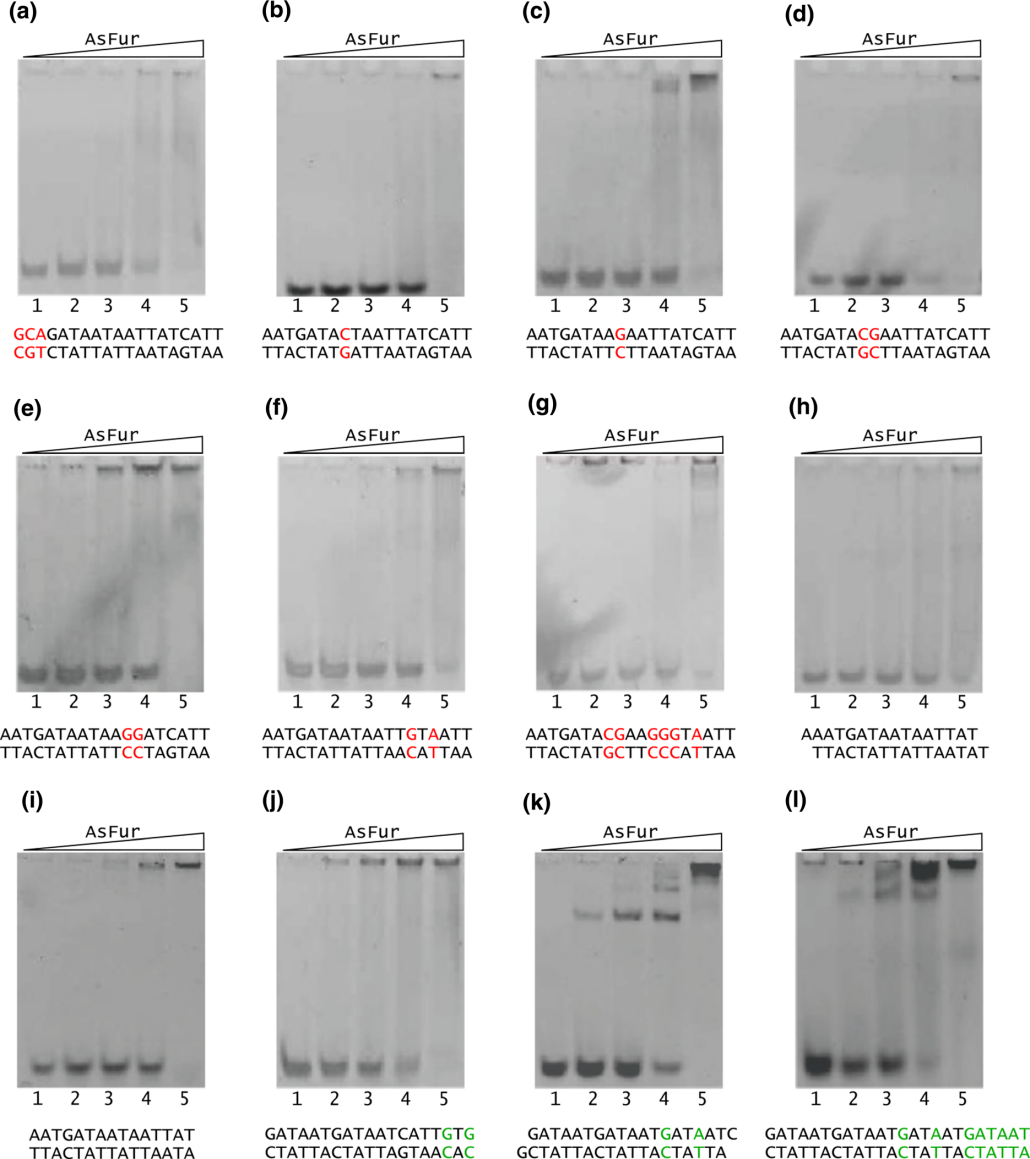
EMSA experiments on variants of Vibrio and *E. coli* consensus oligonucleotides modifying individual positions and/or length. For each experiment, 5 μM DNA was incubated with increasing concentrations of AsFur (0, 10, 20, 40 and 80 μM) for lanes 1–5, respectively. Positions labelled in red or dark green denote modifications with respect to the Vibrio species and *E. coli* consensus sequences, respectively. The experiments were performed in the presence of Mn^2+^. (Color figure online)

Subsequent EMSA experiments were run with oligonucleotides of varying content compared to the two different consensus sequences, in order to investigate the effect of specific base changes on interaction strength with AsFur. The primary targets for the experimental design were the AsFur-DNA interactions predicted by previous MD simulations and binding free energy calculations (Pedersen et al. 2010). In order to further investigate the existing Fur-DNA interaction models, different oligonucleotide lengths (both shorter and longer than the 19 bp Fur box) were probed, as well as variation in the oligonucleotide termini which were either blunt or included a 1 nucleotide overhang capable of forming a ‘sticky’ end with adjacent DNA substrates (Table 1). As expected, the oligonucleotides showed varying interaction strength with AsFur (Fig. 5). For most EMSA experiments, AsFur binding caused the substrate to be retained in the wells of the gel, which most likely reflects the tendency of AsFur to aggregate, possibly triggered by the initial DNA complex formation.

The T12G/T13G substitution reduces interaction of AsFur with DNA considerably (Fig. 5e) compared to the Vibrio consensus sequence (Fig. 4a), suggesting these are key positions for interaction. The corresponding nucleotide positions in the *E. coli* Fur box are T15 and T16 which have previously been shown to interact with *E. coli* Fur by crosslinking experiments (Tiss et al. 2005). Furthermore, molecular dynamics simulations with AsFur indicated T13 as an important contributor in protein interaction (Pedersen et al. 2010). We thus present the first EMSA experiments probing these positions directly in comparison to the Vibrio consensus sequence.

When the A14G/C16A substitution (Fig. 5f) is compared to the Vibrio consensus sequence (Fig. 4a), much reduced interaction capacity with AsFur is observed. These nucleotide positions have previously been shown to contribute favourably to AsFur DNA binding through binding free energy simulations (Pedersen et al. 2010), and the EMSA results further indicate them to participate in sequence-specific interactions. It is interesting to observe that the substitutions A17G/C19G (Fig. 5j) to the *E. coli* consensus sequence (Fig. 4b) also has a detrimental effect, although slightly less pronounced than for the Vibrio consensus sequence. Interestingly, the results highlight the importance of both DNA strands in Fur interaction, as these nucleotide positions form part of the first **G**A**T**AAT hexamer repeat on the complementary strand of both the Vibrio consensus sequence and the *E. coli* Fur box (altered to **T**A**C**AAT and **C**A**C**AAT, respectively).

The A8C and T9G individual substitutions (Fig. 5b, c) also lead to much weaker interactions when compared to the Vibrio consensus sequence (Fig. 4a). AT-rich regions have previously been shown to be essential for Fur-DNA interactions (Hantke 1981, 2001; Prince et al. 1991; Vasil and Ochsner 1999). In particular, the last T base of the GATAA**T** (T_6_) unit in the hexamer repeat model described in Supplementary Figure S2b and c, corresponding to the substituted T9 in the Vibrio consensus sequence, has been highlighted for its role in DNA recognition by footprinting and missing-T assays with *E. coli* Fur (Escolar et al. 1998). The matching T on the complementary strand (T_5_) showed comparable effect in interactions. However, the combined substitutions A8C/T9G (Fig. 5d) does not show an additive effect and has slightly stronger interaction than the individual substitutions. It is interesting that the dual removal of AT-nucleotides in the core of the Vibrio recognition sequence does not appear to further reduce binding strength.

As expected, and in a similar fashion as for the Vibrio least conserved sequence (Fig. 4c) which showed almost no sign of DNA interaction with AsFur, the combined alteration of all nucleotides addressed so far (A8C/T9G/T12G/T13G/A14G/C16A; Fig. 5g) produced a much-weakened interaction with AsFur, although for this EMSA gel some trace amounts of AsFur can be seen shifted to the wells of the gel throughout.

For the A1G/A2C/T3A substitutions (Fig. 5a) compared to the Vibrio consensus sequence (Fig. 4a), substantially reduced interaction strength can be observed. This result is interesting in view of the differences in the 5′-regions of the Vibrio and *E. coli* consensus sequences where the Vibrio consensus sequence has a three-nucleotide ‘insertion’ (AAT) compared to the classical *E. coli* Fur box.

The importance of a minimum length of the Vibrio consensus sequence in AsFur interactions was demonstrated by the EMSA experiments on shortened oligonucleotides compared to the Vibrio consensus sequence (Fig. 5h, i), where both the 16-nucleotide sticky-end variant and the 15-nucleotide blunt-ended oligonucleotides displayed much-reduced binding strength compared to the Vibrio consensus sequence. This agrees well with previous EMSA studies on *E. coli* Fur indicating that only weak interaction is formed when oligonucleotides are considerably shorter than three hexamer repeats of the GATAAT sequence (Lavrrar and McIntosh 2003).

When somewhat similar experiments were performed on GATAAT hexamer repeats of the *E. coli* fur box, either with a 19-nucleotide sticky-end variant (Fig. 5k) or the 24-mer quadruple repeat of the GATAAT sequence (Fig. 5l), reduced interaction strengths were observed for both compared to the *E. coli* Fur box (Fig. 4b). As above, these trends correspond well with EMSA experiments on EcFur with the *E. coli* Fur box, as well as a range of GATAAT repeats, where the interaction strengths were rated as Fur box > 4 × GATAAT > 3 × GATAAT (Lavrrar and McIntosh 2003). The introduction of sticky ends to the triple GATAAT repeat in our study appears to improve binding slightly.

### Analysis of AsFur compared to functional and structural homologs

**Fig. 6:**
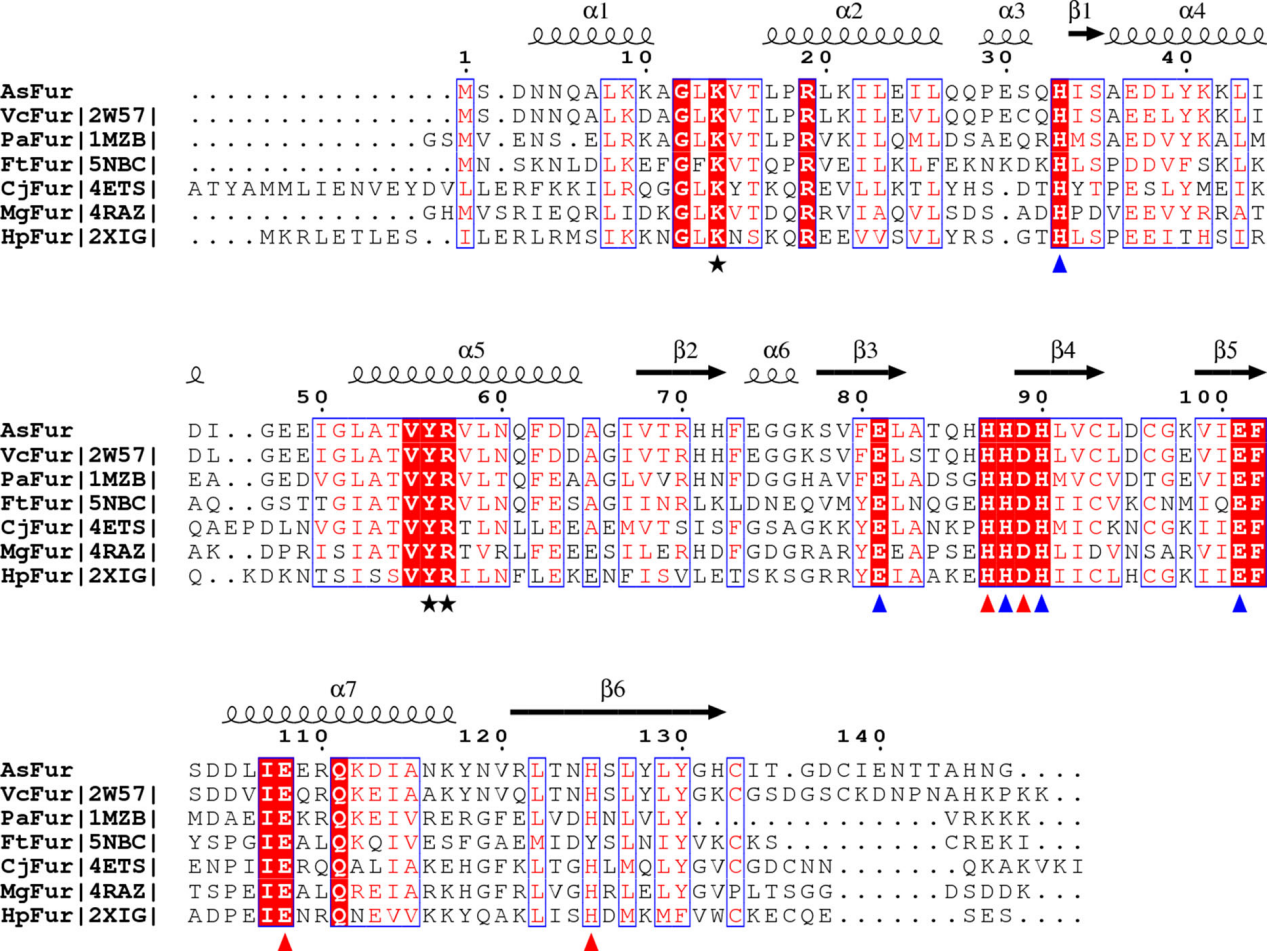
Structure-based sequence alignment of AsFur with known Fur structural homologs. Abbreviations are as defined in Table 2. EcFur was not included as the structure only represents the DBD. PDB identifiers are indicated between vertical lines. Secondary structure elements are shown above the alignment with spirals and arrows indicating α-helices and β-strands, respectively. Identical residues are shown in white on red background, while conserved residues are shown in red. Residues relevant for metal coordination to the regulatory site S2 and structural site S3 are indicated with triangles (coloured blue and red for S2 and S3, respectively), while residues forming base contacts are indicated with a black asterisk. (Color figure online)

To enable analysis of structural interactions contributing to specificity of binding between AsFur and variations on canonical Fur-box sequences, the sequence of AsFur was compared to structurally-determined homologs. A number of structurally-characterized homologs of AsFur were identified with sequence identities ranging from 86 to 30% (Table 2 and Fig. 6). The sequence alignment between these homologs and AsFur highlights several conserved sequence patches both within the DNA-binding- and dimerization domains (DBD and DD, respectively). Although most Fur proteins characterised are found to be dimers in solution, some also exist in the form of stable tetramers, exemplified by Fur from *Francisella tularensis* and *Pseudomonas aeruginosa* (Nader et al. 2019; Perard et al. 2018). While the conserved positions in the DD are mainly attributed to metal-coordination, the conserved patches in the DBD are involved in interactions with the Fur box (Fig. 6).

**Table 2:** Comparison of AsFur with known Fur structural homologs

Abbreviation	Species	PDB	#Amino acids	#Identical/#aligned	Seq. id. (%)	References
AsFur	*Aliivibrio salmonicida*	Model	147	–	–	
EcFur	*Escherichia coli* (DBD)	2fu4	83	71/83	86	Pecqueur et al. (2006)
VcFur	*Vibrio cholerae*	2w57	150	125/146	86	Sheikh and Taylor (2009)
PaFur	*Pseudomonas aeruginosa*	1mzb	136	60/126	75	Pohl et al. (2003)
FtFur	*Francisella tularensis*	5nbc	140	52/133	39	Perard et al. (2018)
CjFur	*Campylobacter jejuni*	4ets	162	53/133	40	Butcher et al. (2012)
MgFur	*Magnetospirillum Gryphiswaldense Msr-1*	4raz/4rb1/4rb3	145	51/135	37	Deng et al. (2015)
HpFur	*Helicobacter pylori*	2xig	150	41/137	30	Dian et al. (2011)

**Fig. 7:**
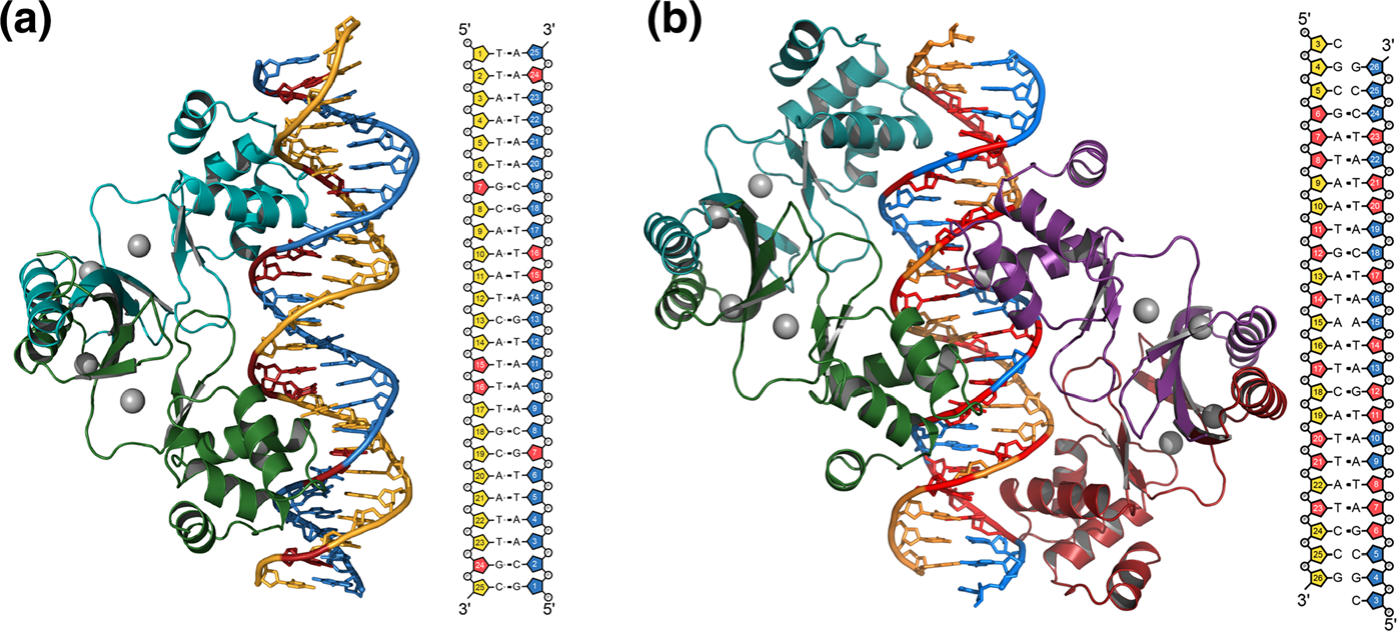
Homology models of AsFur in the two Fur-DNA interaction modes observed for MgFur and reported by Deng et al. (2015). **a** A dimer of AsFur interacting with the *feoAB1* operator. **b** Two AsFur dimers interacting with the *E. coli* Fur box. Each AsFur monomer is coloured individually (monomer A, dark green; monomer B, turquoise; monomer C, purple; monomer D, red) and the DNA strands are coloured in dark yellow and blue for the primary and complementary strands, respectively. Nucleotides coloured in red indicate base contacts with AsFur. The modelled Mn^2+^ ions are indicated as grey spheres. (Color figure online)

Based on the sequence identity between *V. cholerae* Fur (VcFur) and AsFur at 86%, the crystal structure of VcFur (Sheikh and Taylor 2009) would be a preferred choice for homology modelling. However, this crystal structure represents Fur in an unbound state and structural alignments between the unbound and DNA-bound states of the MgFur structures revealed substantial movements in the DBD region upon DNA binding with VcFur and the MgFur DNA complexes having root-mean-square deviations of 2.6–3.0 Å. Thus, for homology modelling, the published structures of MgFur (Deng et al. 2015) were selected as templates despite the relatively low sequence similarity with AsFur. Sequence alignment between AsFur and MgFur revealed 37% identity for the 135 residues that could be structurally aligned and enabled reliable modelling of the entire protein including the N-terminal DNA-binding domain (DBD), which is highly flexible in the un-bound form of MgFur (Deng et al. 2015; Sarvan et al. 2018). To compare different possible binding modes of AsFur, two models were constructed: the first based on the MgFur dimer bound to a *feoAB1* operator as a 9-1-9 inverted repeat (PDB4rb3; Supplementary Figure S2a) and the second based on the two MgFur dimers bound to an *E. coli* Fur box as a 7-1-7 inverted repeat offset by 6 nucleotides (PDB4rb1; Supplementary Figure S2e). Comparison of these models reveals a conservation in amino acids in the interacting regions of the two proteins (Fig. 7).

### Analysis of structural determinants of AsFur-DNA interaction

The homology models generated based on MgFur were analysed to structurally rationalize the variations in interaction strengths from EMSA.

**Fig. 8:**
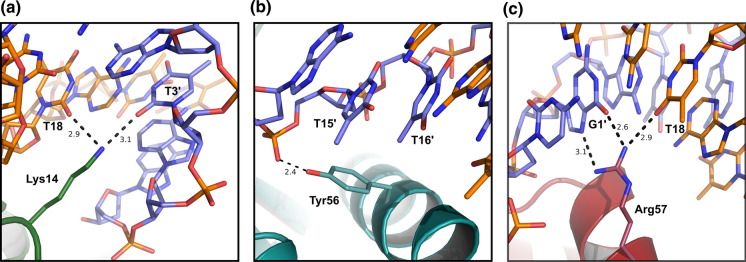
Predicted AsFur-nucleotide base interactions from the homology model. Nucleotide base-interactions observed in the AsFur homology model based on the crystal structure of two dimers of MgFur in complex with the *E. coli* Fur box (PDB4rb1). Nucleotide numbering follows the numbering scheme used for the *E. coli* consensus sequence in Table 1. **a** Lys14 in monomer A interacts in the minor groove with T18 on the primary strand and T3′ on the complementary strand. **b** Tyr56 in monomer B forms hydrophobic interactions in the major groove with T15′ and T16′ on the complementary strand (identical interactions are formed between Tyr56 in monomer D, generated through a crystallographic symmetry operation, and T15/T16 on the primary strand). **c** Arg57 in monomer D interacts in the major groove with T18 on the primary strand and G1′ on the complementary strand. (Color figure online)

These strongly indicate that AsFur Tyr56 forms base-specific major groove interactions through hydrophobic interactions with the methyl groups of both T12 and T13 (Fig. 8b), explaining the observed decrease in binding affinity in the T12G/T13G substitution. Interestingly, this interaction is conserved in both structural models, i.e. both in the forms of a 9-1-9 inverted repeat, as well as in the 7-1-7 inverted repeat offset by 6 nucleotides, highlighting the role of Tyr56 in interactions with Fur box-containing DNA.

The homology model of AsFur interacting with the *E. coli* Fur box (Fig. 7b) also highlights the importance of both DNA strands in this interaction, providing a rationale for the impact of the A17G/C19G substitution (Fig. 5j) with nucleotide base-contacts formed in the 5′-end of the complementary strand. The detailed view of the interactions shown in Fig. 8a, c, illustrates that the nucleotide positions in the first hexamer repeat of the complementary strand (G1′ and T3′) form minor- and major-groove nucleotide base interactions with the conserved residues Lys14 and Arg57, respectively. Arg57 appears to form bidentate base-specific major-groove interactions, while the minor-groove interactions formed by Lys14 are base-unspecific. The corresponding Lys residue in MgFur has previously been shown to interact with the DNA target through a shape readout mechanism, where the AT-rich region in each hexamer repeat results in a narrow minor groove with enhanced negative electrostatic potential (Deng et al. 2015). Previous studies have indicated that most DNA-binding proteins use interplay between the base- and shape-readout modes to recognize their DNA binding sites (Slattery et al. 2014). This in turn allows for alterations in the specific nucleotide succession, and for the specific case of Fur, thus rationalises the degree of degeneracy found among Fur recognition sequences.

While Lys14, Tyr56 and Arg57 are also found to interact with nucleotide bases in the homology model of AsFur in complex with the *feoAB1* operator (Fig. 7a), these interactions can not to the same extent justify a structural rationalisation of the above-mentioned effects from our EMSA experiments, making it less likely that AsFur interacts with the Vibrio consensus sequence and the *E. coli* Fur box as one dimer in the form of an 9-1-9 inverted repeat.

The AsFur homology model with the *E. coli* Fur box does not show direct contacts in the 5′-region of the Vibrio consensus sequence (upstream of the first GATAAT repeat), which is equivalent to the position of the substitution A1G/A2C/T3A; however, it is likely that Lys14 from the DBD of monomer B may undergo conformational changes in order to be involved in minor groove interactions. In fact, previous studies have suggested that the Fur box should be extended in the 5′-end, where Baichoo et al. (Baichoo and Helmann 2002) suggested an additional T extension in the *B. subtilis* Fur box and Chen et al. (Chen et al. 2007) that the *E. coli* Fur box should include the sequence AAT, i.e. identical to the Vibrio consensus sequence.

## Conclusion

Fur has an important role in iron homeostasis and regulation of virulence mechanisms in many pathogenic bacteria. In an attempt to better understand the molecular basis behind DNA-recognition by AsFur, we have examined its DNA interaction with the combined use of interaction assays and structural modeling, which allowed for a structure/function interpretation of the biochemical results obtained.

AsFur was found to be a dimer during purification conditions. Due to protein instability issues, it was difficult to further investigate the stoichiometric rates on its interaction with different consensus sequences. However, the combined output of the homology modelling and the EMSA investigations indicate that AsFur will be able to interact in the form of two dimers.

The combined results of the EMSA experiments and homology models indicate that AsFur binding strength to DNA is stronger for longer oligonucleotides than shorter, and we observed a small increase in binding strength when sticky ends were introduced to the same oligo sequence. The results further showed that no single base mutations were crucial, and that only anti-consensus depleted binding completely. However, nucleotide positions T12 and T13 (T15 and T16 in *E. coli*) and A14 and C16 (A17 and T19 in *E. coli*) previously suggested to be in direct contact with Fur, lead to a markedly reduced binding strength between AsFur and DNA when mutated. This indicated that these bases were important for AsFur-DNA specific interaction. In addition, mutations of individual and dual AT bases in the core of the vibrio consensus sequence highlighted the importance of AT-rich regions for interaction with AsFur.

The interplay between base- and shape-readout modes, allowing degeneracy between Fur consensus sequences within and between bacteria, was also for AsFur important in binding site recognition. Similarity in Fur-DNA interaction mode between bacteria through base readout by conserved Tyrosine and Arginine residues and shape readout by conserved Lysine residue.

In summary, biochemical assays combined with structural modeling has provided further insight into the AsFur-DNA interaction mode.

## Electronic supplementary material

Below is the link to the electronic supplementary material.

Supplementary file1 (DOCX 857 kb)
