# Supplementary material for: Biochemical characterization of ferric uptake regulator (Fur) from Aliivibrio salmonicida. Mapping the DNA sequence specificity through binding studies and structural modelling
Source: Biometals. 2020 Jul 9;33(4):169–85. doi: 10.1007/s10534-020-00240-6 (PMC7536154; doi:10.1007/s10534-020-00240-6)
Supplement: Supplementary file 1 — Supplementary file1 (DOCX 857 kb) [file 10534_2020_240_MOESM1_ESM.docx]

**Title**: Biochemical characterization of ferric uptake regulator (Fur) from *Aliivibrio salmonicida*. Mapping the DNA sequence specificity through binding studies and structural modelling.

**Authors**: Kristel Berg, Hege Lynum Pedersen and Ingar Leiros*

The Norwegian Structural Biology Centre (NorStruct), Department of Chemistry, Faculty of Science and Technology, UiT the Arctic University of Norway, N-9037 Tromsø, Norway.

***Correspondence:** Ingar Leiros, The Norwegian Structural Biology Centre (NorStruct), Department of Chemistry, Faculty of Science and Technology, UiT the Arctic University of Norway, N-9037, Tromsø, Norway. **Phone**: (+47) 95966895 **Email**: [ingar.leiros@uit.no](mailto:ingar.leiros@uit.no)

**ORCID ID:** Kristel Berg: 0000-0001-9951-9934; Hege Lynum Pedersen: 0000-0002-3768-9513; Ingar Leiros: 0000-0001-9333-4541

**
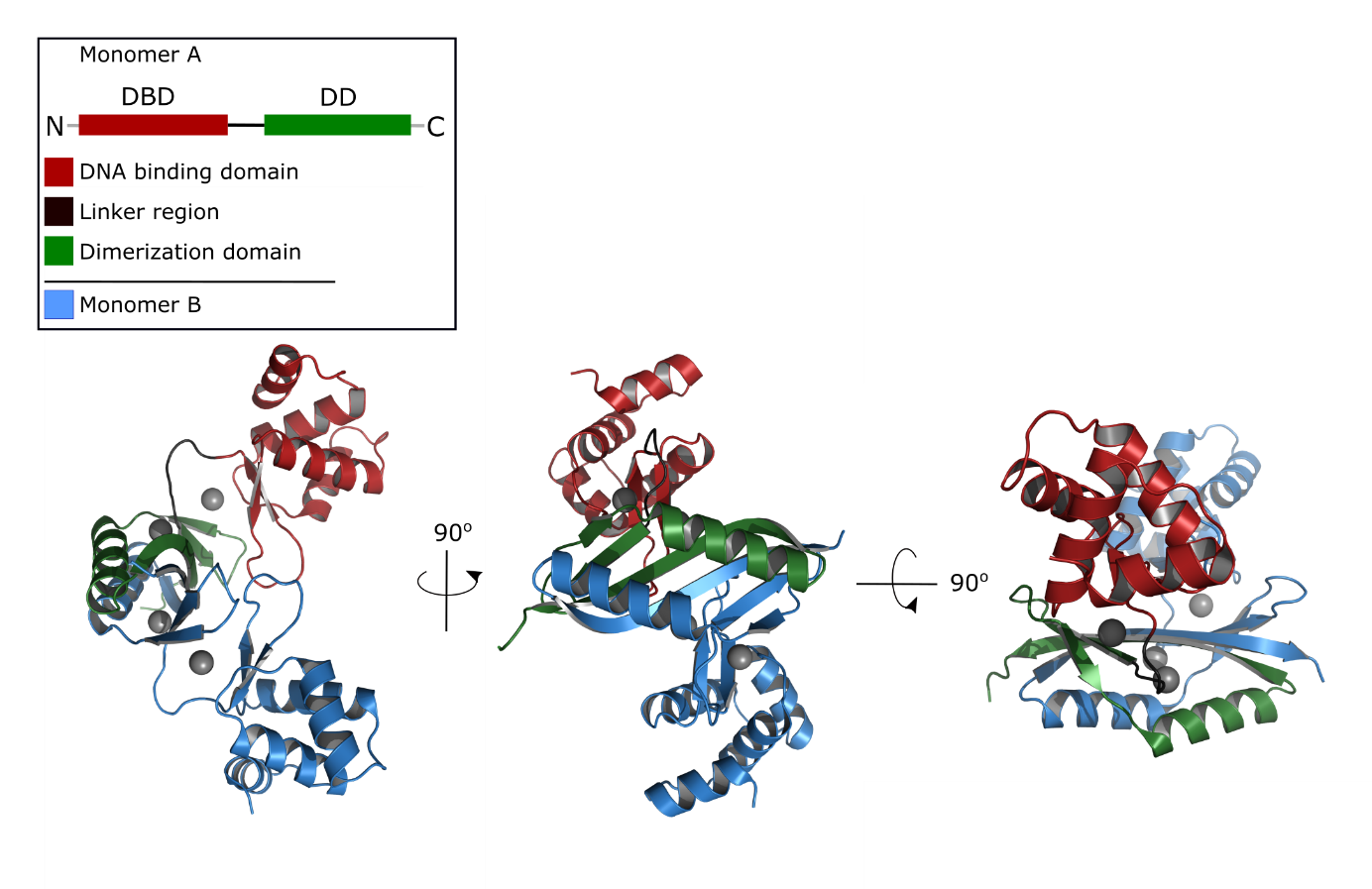
**

**Supplementary Figure 1.** Cartoon representations of dimeric Fur in three different rotated views. Grey spheres represent Mn^2+^-ions. Otherwise, colour codes are as described in the inset. The figure is based on the published structure of MgFur (PDB4rb3, not showing DNA; (Deng et al. 2015)).

**Supplementary text:**

The classical Fur box (Supplementary Figure 2a) originates from DNase I protection- and footprinting-experiments on *E. coli* Fur, where a Fur dimer recognizes a 19 bp inverted repeat sequence: 5’-GATAATGATAATCATTATC-3’ (Escolar et al. 1999), although this exact sequence is not found in the *E. coli* genome. This inverted repeat operator site was confirmed by binding of Fur to oligonucleotides inserted into a plasmid (Calderwood and Mekalanos 1988). In addition, Fur boxes from other genera have also been characterized and described (Baichoo and Helmann 2002; Escolar et al. 1998; Escolar et al. 1999; Escolar et al. 2000; Pedersen et al. 2010; Pich et al. 2012).

Further studies showed that Fur protects a region somewhat larger than the Fur box, indicating that Fur interacts with DNA also outside the Fur box region. Several Fur dimers may bind two or more overlapping Fur boxes, even extended up to ~100 bp (Escolar et al. 2000), as Fur appears to polymerize along the DNA from the initial primary site to weaker secondary sites. New insights to the binding region led to a revised model, suggesting that Fur recognizes three repeated arrays of GATAAT in *E. coli* with a slight imperfection in the third array, rather than the classical palindromic 19 bp sequence (Lavrrar and McIntosh 2003). In that case, dimeric Fur is suggested to interact with the AT-AT-repeat (G**AT**A**AT**) within each hexamer, with two overlapping dimers binding. Supplementary Figure 2b illustrates how the GATAAT hexamer can be interpreted as three direct repeats in a tandem array F-F-F fashion, although the last hexamer could be inverted to an F-F-R arrangement or have mismatches. Alternatively, the hexamers can be arranged as two direct repeats, followed by an inverted repeat, separated by a single bp, as illustrated by the hexamer model F-F-x-R in Supplementary Figure 2c. The latter organization has been shown to appear most frequently in natural Fur binding sites and also has the highest affinity for Fur (Escolar et al. 1998). A revised model of how Fur interacts with its target was suggested by Lavrrar *et al*, who predicted the minimum Fur box as a 13 bp overlapping 6-1-6 motif (Supplementary Figure 2d) with two Fur dimers interacting from opposite faces of the helix, explaining the corkscrew manner Fur wraps around the DNA duplex (Lavrrar et al. 2002). A similar reinterpretation of the Fur box, based on alignment of *Bacillus subtilis* Fur boxes and DNase footprinting, proposes a slightly longer Fur box core sequence consisting of two overlapping heptamer inverted repeats (Baichoo and Helmann 2002; Baichoo et al. 2002). These two 7-1-7 motifs generate a slightly extended Fur consensus sequence of 21 bp (Supplementary Figure 2e). Crystal structures determined for complexes of DtxR bound to its operator site show a similar binding model (Pohl et al. 1999; White et al. 1998).


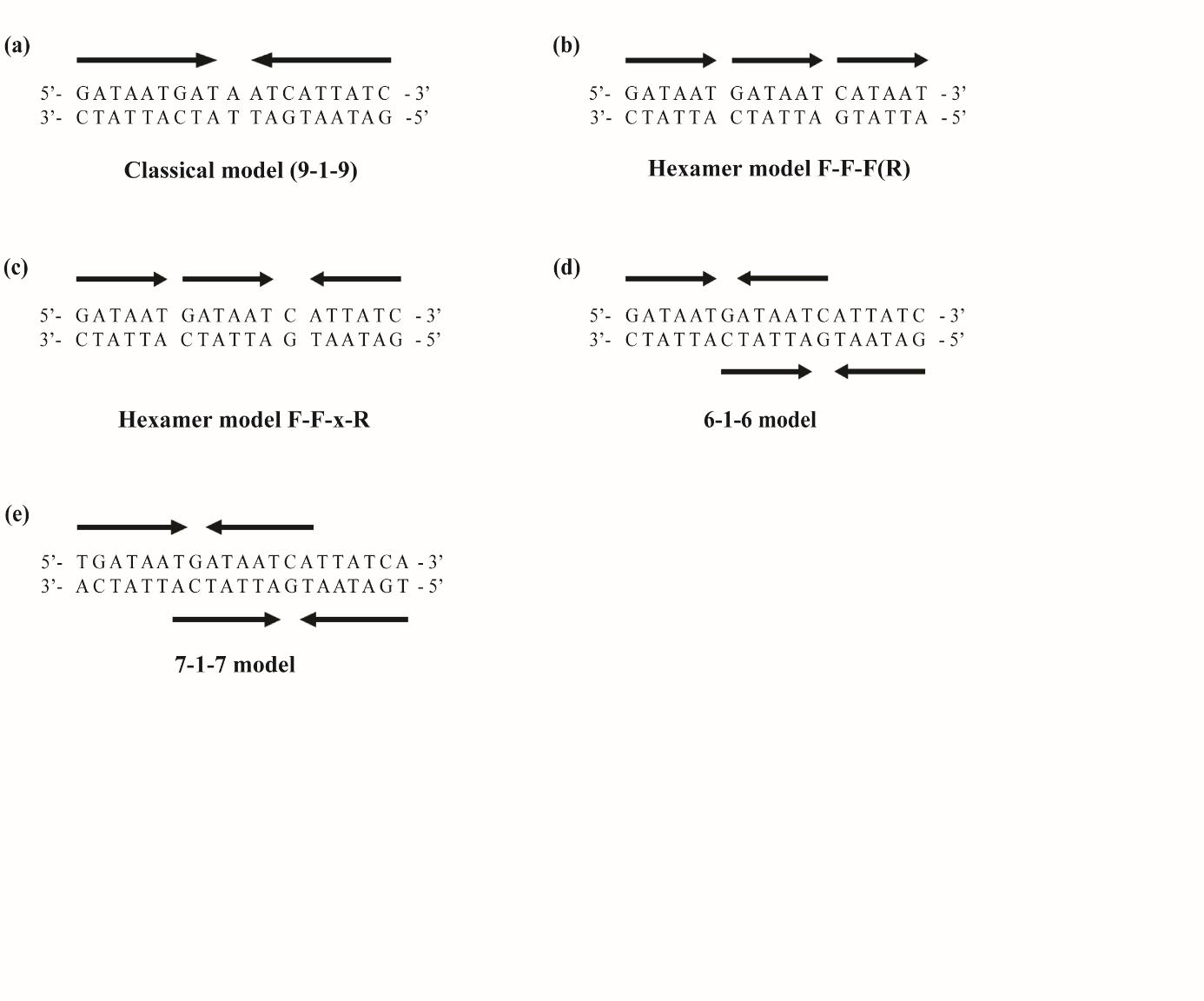


**Supplementary Figure 2. Alternative arrangements of the Fur box, illustrated by the *E. coli* consensus with GATAAT as the minimal recognition unit (Fur consensus NATA/TAT).** Arrows mark inverted repeats/repeated arrays. **a**) The 19 bp classical model suggests two inverted repeats with an A:T basepair in between, binding a monomer each (Escolar et al. 1999). **b**) The 18 bp hexamer model contains a minimum of three direct repeats of the hexamer GATAAT, where the AT-AT pattern within each hexamer was suggested to interact with Fur. The last hexamer may be reversed or imperfect (Lavrrar and McIntosh 2003). **c**) The 19 bp hexamer model is described as repeated arrays of three or more copies of GATAAT motifs, recognized by two hexamers in the forward direction and one hexamer at the reverse orientation, separated by one base pair (Escolar et al. 1998). **d**) The hexamer model can be viewed as a 6-1-6 arrangement, where two overlapping hexamer inverted repeats binds Fur dimers at opposite faces of the double helix (Lavrrar et al. 2002). **e**) The 21 bp 7-1-7 model defines the Fur box as two overlapping heptamer inverted repeats, also recognized by two Fur dimers at opposite faces (Baichoo and Helmann 2002; Baichoo et al. 2002).


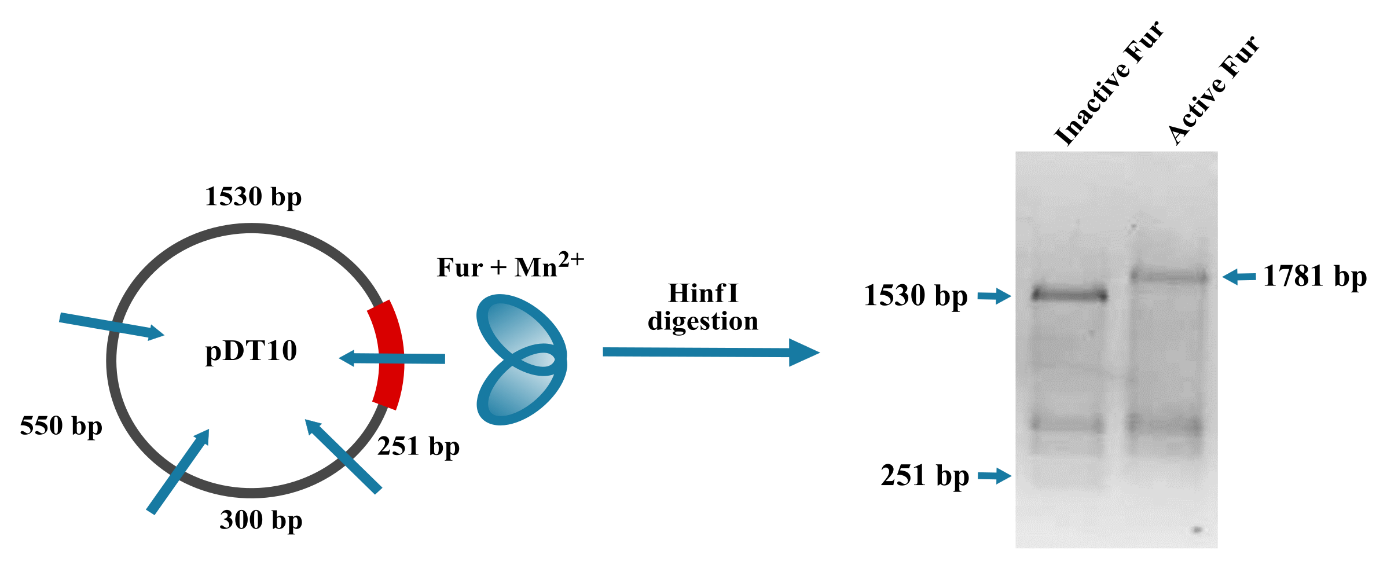
 **Supplementary Figure 3. Plasmid protection assay**. Active Fur dimer binds the incorporated *E. coli* Fur box consensus in the pDT10 plasmid and protects the specific site from HinfI digestion. The DNA protection due to Fur activity is visible as an altered migration pattern on a 1 % agarose gel. The Fur binding site is highlighted in red and HinfI restriction sites are marked by blue arrows, also including expected fragment sizes.

**References:**

Baichoo N, Helmann JD (2002) Recognition of DNA by Fur: a reinterpretation of the Fur box consensus sequence. J Bacteriol 184:5826-5832

Baichoo N, Wang T, Ye R, Helmann JD (2002) Global analysis of the Bacillus subtilis Fur regulon and the iron starvation stimulon. Mol Microbiol 45:1613-1629

Calderwood SB, Mekalanos JJ (1988) Confirmation of the Fur operator site by insertion of a synthetic oligonucleotide into an operon fusion plasmid. J Bacteriol 170:1015-1017

Deng Z et al. (2015) Mechanistic insights into metal ion activation and operator recognition by the ferric uptake regulator. Nat Commun 6:7642 doi:10.1038/ncomms8642

Escolar L, Perez-Martin J, de Lorenzo V (1998) Binding of the fur (ferric uptake regulator) repressor of Escherichia coli to arrays of the GATAAT sequence. J Mol Biol 283:537-547 doi:10.1006/jmbi.1998.2119

Escolar L, Perez-Martin J, de Lorenzo V (1999) Opening the iron box: transcriptional metalloregulation by the Fur protein. J Bacteriol 181:6223-6229

Escolar L, Perez-Martin J, de Lorenzo V (2000) Evidence of an unusually long operator for the fur repressor in the aerobactin promoter of Escherichia coli. The Journal of biological chemistry 275:24709-24714 doi:10.1074/jbc.M002839200

Lavrrar JL, Christoffersen CA, McIntosh MA (2002) Fur-DNA interactions at the bidirectional fepDGC-entS promoter region in Escherichia coli. J Mol Biol 322:983-995

Lavrrar JL, McIntosh MA (2003) Architecture of a fur binding site: a comparative analysis. J Bacteriol 185:2194-2202

Pedersen HL et al. (2010) Experimental and computational characterization of the ferric uptake regulator from Aliivibrio salmonicida (Vibrio salmonicida). J Microbiol 48:174-183 doi:10.1007/s12275-010-9199-5

Pich OQ, Carpenter BM, Gilbreath JJ, Merrell DS (2012) Detailed analysis of Helicobacter pylori Fur-regulated promoters reveals a Fur box core sequence and novel Fur-regulated genes. Mol Microbiol 84:921-941 doi:10.1111/j.1365-2958.2012.08066.x

Pohl E, Holmes RK, Hol WG (1999) Crystal structure of a cobalt-activated diphtheria toxin repressor-DNA complex reveals a metal-binding SH3-like domain. J Mol Biol 292:653-667 doi:10.1006/jmbi.1999.3073

White A, Ding X, vanderSpek JC, Murphy JR, Ringe D (1998) Structure of the metal-ion-activated diphtheria toxin repressor/tox operator complex. Nature 394:502-506 doi:10.1038/28893
